# Air Pollution in Kazakhstan and Its Health Risk Assessment

**DOI:** 10.5334/aogh.2535

**Published:** 2019-11-08

**Authors:** D. Kenessary, A. Kenessary, Z. Adilgireiuly, N. Akzholova, A. Erzhanova, A. Dosmukhametov, D. Syzdykov, Abdul-Razak Masoud, Timur Saliev

**Affiliations:** 1 “Kenesary Company” LLP, Almaty, KZ; 2 National Laboratory Astana, Nazarbayev University, Astana, KZ; 3 B. Atchabarov Scientific Research Institute of Fundamental Medicine, Almaty, KZ

## Abstract

**Background:**

Air pollution in Kazakhstan is caused by many factors and poses serious threats to public health. Ambient air in the cities of Kazakhstan is polluted due to mining and processing of mineral resources, oil and gas production, gasoline and diesel fuel motor vehicles, industrial enterprises.

**Objective:**

The study aim is to assess the air pollution degree in most significant settlements of Kazakhstan and define risk levels for the population health. Ambient air monitoring was conducted in 26 cities. Air pollution severity was assessed by the analysis results and processing of air samples taken at the stationary observation posts. Health risk assessment due to chemical factors was calculated according to the approved risk assessment methodology.

**Findings:**

There is high risk of acute adverse effects risk from suspended particles, oxides and dioxides of nitrogen and sulfur in almost all of the studied cities. The most unfavorable situation is in Ust-Kamenogorsk. Also, there is the adverse chronic effects risk caused by suspended particles exposure in majority of the studied cities. Extremely high chronic effects risk as a result of heavy metals exposure was detected in Ust-Kamenogorsk, Shymkent, Almaty, Taraz and Balkhash. Unacceptable carcinogenic risk levels have been determined for professional groups and the whole population with respect to cadmium in Shymkent, Almaty, Balkhash; arsenic in Shymkent, Almaty, Balkhash; lead in Taraz; chromium – in Shymkent, Aktobe, Almaty and Balkhash. Thus, the values of the hazard quotients and indices for acute and chronic exposure in most of the studied cities of Kazakhstan exceed the permissible level equal to 1.0.

**Conclusion:**

Due to the unacceptable risk levels in the cities it is strongly recommended to conduct a detailed study of the health status of the population depending on the air pollution.

## Introduction

Exposure of the populace to ambient air pollution has been considered as a significant contributor to the development of a range of disorders [2,15]. In fact, polluted air is still a substantial threat to people’s health around the world, despite the introduction of new technologies in industry, energy and transportation [3,4,10,13].

A number of works demonstrated pollution of atmospheric air as the primary environmental factor that causes a high level of health risk in urbanized areas [6,7,8,14,16]. Nowadays, the air basin of almost any settlement is polluted with hundreds of chemical substances, the level of which, as a rule, exceeds the maximum permissible threshold, and its combined effect is even more significant [1,5,11,12].

Taking into account the impact of pollution on public health, this study aims to assess the air pollution level in all settlements of the Republic of Kazakhstan according to the information bulletins based on the data provided by KAZHYDROMET—the regional state enterprise responsible for monitoring and analyzing the environmental situation in the Republic of Kazakhstan.

In fact, air pollution in Kazakhstan is caused by many factors. First on the list is the recent growth of mining and processing of mineral resources, such as lead, zinc, phosphorus, and chromium productions. Mining produces a huge volume of waste. 20 billion tons of this waste is accumulated and a third of them contaminate the air on a daily basis. Domestic mining enterprises use old, inefficient purification systems, as a result of which tons of harmful substances are released into the atmosphere.

The second cause of air pollution is flaring of gas during oil and gas production. This is accompanied by soot emissions. Instead of utilizing the gas, the producers found it cheaper to burn it out, thus contributing to the pollution of the air with carbon dioxide.

Another main contributor to air pollution is gasoline and diesel fuel motor vehicles. The increased number of cars, particularly in the main cities of Kazakhstan, results in a high level of air pollution by nitrogen dioxide, carbon monoxide, and organic substances.

The next factor is the dispersion of emissions from industrial enterprises as the result of production processes during industrial products combustion. In fact, there is the entire list of harmful substances causing the high level of air pollution in Kazakhstan. Pollutants dispersion in the air basin over the territory of settlements significantly affects the atmospheric air quality of cities, suburbs and towns.

All of the above-mentioned problems deteriorated, owing to issues with air ventilation in main cities, due to the bad architecture planning or specifics of landscapes. Inadequate airing of the atmospheric space in settlements leads to pollutants accumulation in the surface atmosphere layer, and their concentrations remain at very high levels. As a consequence of these factors, the permissible level of air pollution is exceeded in 13 major cities of Kazakhstan (Ust-Kamenogorsk, Aktobe, Astana, Almaty, Petropavlovsk, Atyrau, Balkhash, Shymkent, Temirtau, Zhezkazgan, Taraz, Karaganda, Semey cities).

## Materials and methods

Monitoring of atmospheric air pollution was conducted in 26 settlements in the Republic of Kazakhstan, at 146 observation posts to be specific, including 56 stationary posts. There are three programs of the atmospheric air quality observation: complete, incomplete and short. The complete air observation program is intended to receive information about single and daily average concentrations. In this case, observations are performed daily by continuous registration using automatic devices or discretely at regular intervals. The measurements are carried out at least four times a day with mandatory sampling at 1, 7, 13, 19 o’clock local time. The incomplete observation program is carried out to obtain information about single concentrations daily at 7, 13 and 19 o’clock of local time. The short observation program is carried out in order to obtain information only about single concentrations every day at 7 and 13 o’clock local time.

The extent of air pollution was assessed by the results of analysis and processing of air samples taken at the observation posts.

The following indicators were monitored at the observation posts in order to reveal the extent of the air pollution: suspended particles (dust), suspended particles PM-2.5, suspended particles PM-10, sulfur dioxide, soluble sulfates, carbon dioxide, carbon monoxide, nitrogen dioxide, oxide nitrogen, ozone (surface), hydrogen sulfide, phenol, hydrogen fluoride, chlorine, hydrogen chloride, hydrocarbons, ammonia, sulfuric acid, formaldehyde, methane, inorganic arsenic compounds, cadmium, lead, chromium, copper, benzene, benzapyrene, beryllium, manganese, cobalt, gamma background radiation, zinc.

Health risk assessment due to chemical factors, particularly from chemical substances contained in atmospheric air, was calculated according to the “Guidelines for the public health risk assessment when exposed to chemical substances that pollute the environment.” This is the manual for the population health risk assessment due to chemical substances exposure that pollutes the environment (P 2.1.10.1920-04), approved by the Chief State Sanitary Doctor of the Russian Federation (05.03.2004). It is based on the risk assessment methodology previously developed by the United States Environmental Protection Agency (US EPA) [9,17]. The following reference values were used for risk assessment (Table 1).

**Table 1:** Referent values of pollutants in ambient air of populated areas.

Substances	Referent values (mg/m^3^)	

	Maximum single	Average daily

Ammonia	0.2	0.04
Benz(a)pyrene	–	0.1 mkg/100 m^3^
Suspended particles (dust)	0.5	0.15
Suspended particles PM-10	0.3	0.06
Suspended particles PM-2.5	0.16	0.035
Hydrogen fluoride	0.01	0.001
Nitrogen dioxide	0.2	0.04
Sulfur dioxide	0.5	0.05
Copper	0.003	0.002
Cadmium	–	0.0003
Manganese	0.01	0.001
Methane	46.7	10
Arsenic	0.04	0.01
Ozone (ground level)	0.16	0.03
Nitrogen oxide	0.4	0.06
Carbon monoxide	5.0	3.0
Hydrogen sulfide	0.008	–
Lead	0.001	0.0003
Sulfates	0.003	0.002
Phenol	0.01	0.003
Formaldehyde	0.05	0.01
Chlorine	0.1	0.03
Hydrogen chloride	0.2	0.1
Hydrogen fluoride	0.02	0.005
Chromium	–	0.0015
Zinc	–	0.05

According to this methodology, non-carcinogenic risk assessment was carried out based on the calculation of hazard quotient (HQ), using the formula:

{\rm{HQ}}\, = \,{{\rm{C}}_{{\rm{actual}}}}{\rm{/RfC}},

where C – actual concentration of the substance in the air;

RfC – reference concentration.

If HQ is equal to or less than 1.0, the risk of being subjected to harmful effects is considered extremely low, and with an increase in the HQ quotient, the probability of adverse effects occurring increases, i.e. HQ > 1.0 is considered as evidence of potential health risks.

Risk assessment of the non-carcinogenic effects development from a combined exposure of chemical compounds was carried out on the basis of hazard index calculation (HI) for simultaneous intake of several substances in the same way (inhalation). Hazard indices were calculated for substances affecting the respiratory system. The permissible value of the hazard index is no more than 1. Even if HQ of particular substances is less than 1, HI may exceed 1. Calculations of hazard indices were carried out according to the following formula:

{\rm{HI}}\, = \,\Sigma\ {\rm{ HQi,}}

where HQi – hazard quotients of particular chemical substances.

For non-carcinogenic chemical substances, additivity is confirmed if they have the same (homogeneous) toxic effect. In accordance with international recommendations, the “same” action conditionally means the effect of substances on the same organs or systems.

The risk assessment for the development of carcinogenic effects was evaluated using the individual carcinogenic risk concept. Individual carcinogenic risk is an assessment of the probability of cancer development in an affected individual exposed to potential carcinogens throughout his/her lifetime (the average life expectancy is assumed to be 70 years). It is assumed that all identified carcinogens affect the individual throughout life.

Individual carcinogenic risk (ICR) was estimated using the following formula:

{\rm{ICR}}\, = \,{\rm{LADD}}\, \times \,{\rm{SF,}}

where LADD – life average daily dose, mg/(kg*day)

{\rm{LADD }} = {\rm{ AC}}\left({\rm{D}} \right){\rm{ }} \times {\rm{ 20/70,}}

where AC – average daily/annual concentration/dose, mg/m^3^

Seventy (70) kg is the average human weight and 20 m^3^ is the average daily air consumption.

The ICR indicator describes the individual risk of malignant neoplasms in a hypothetical person exposed to the studied factor (chemical substance).

In assessing the carcinogenic risk, as a rule, only the chronic effects of substance are taken into account, i.e. annual/daily average concentrations are used.

According to the risk assessment methodology, there are criteria for the acceptability or admissibility of the carcinogenic risk, both for professional groups and for the whole population. According to the classification of the carcinogenic risk levels, there are four ranges of its acceptability. Thus, the first range includes the individual risk (ICR) throughout life, equal to or less 1 × 10^–6^, which corresponds to one additional case of cancer per 1 million exposed people. This range characterizes such risk levels that are perceived by all as negligible, not different from ordinary, everyday risks. Such cases do not require any additional measures to reduce them, but their levels need periodic monitoring.

The second range (ICR more 1 × 10^–6^, but less 1 × 10^–4^) corresponds to the maximum permissible risk, i.e. upper limit of acceptable risk. At this level, most of the hygienic standards recommended by foreign and international organizations for the whole population are determined (for example, WHO uses the acceptable risk value for drinking water equal to 1 × 10^–5^, for the atmospheric air – 1 × 10^–4^). These levels need constant monitoring.

The third range (ICR more 1 × 10^–4^, but less 1 × 10^–3^) is acceptable for professional groups but not for the whole population. The emergence of such risk requires the development and implementation of planned sanitation activities.

Fourth range (ICR ≥ 1 × 10^–3^) is not acceptable for both the population and professional groups. In this case, it is necessary to implement emergency sanitary measures so as to reduce the risk.

## Results

This study analyzed the quality of atmospheric air in the main cities of Kazakhstan in context of its impact on the health of the populace. Hazard quotients were calculated separately for every substance at each calculated point, then scaled for different conditions (acute and chronic effects).

When calculating the hazard quotient of acute exposure (HQ acute, Table 2) the maximum single concentrations of the main pollutants in the atmospheric air of the studied cities were taken into account according to the official data the official data of KAZHYDROMET regional state enterprise (for 2017).

**Table 2:** Acute exposure hazard quotients (HQ acute) caused by the main chemical pollutants of atmospheric air in the studied cities of the Kazakhstan. 1 – Shymkent. 2 – Astana. 3 – Kokshetau. 4 – Stepnogorsk. 5 – Borovoye. 6 – Shchuchinsk-Borovoy resort area. 7 – Aktobe. 8 – Almaty. 9 – Taldykurgan. 10 – Atyrau. 11 – Ust-Kamenogorsk. 12 – Semey. 13 – Taraz. 14 – Uralsk. 15 – Aksay. 16 – Karaganda. 17 – Balkhash. 18 – Zhezkazgan. 19 – Temirtau. 20 –Kostanay. 21 – Kyzylorda. 22 – Aktau. 23 –Pavloda. 24 – Ekibastuz. 25 – Petropavlovsk. 26 – Turkestan.

*Cities	1	2	3	4	5	6	7	8	9	10	11	12	13	14	15	16	17	18	19	20	21	22	23	24	25	26

Substances																										

Ammonia	1.1			0.3	0.6	0.5	0.9		1.0	0.1	0.3	0.6	0.1	0.2	0.6	0.2	0.2	0.6	1.3			0.2	0.6	0.4	0.4	
Suspended particles (dust)	2.3	14.7	3.7		0.7	1.7	1.3	2.3	3.3	4.0	4.7	2.0	7.0			2.3	8.3	7.0	3.7	4.0	3.3	1.7	3.3	2.7	0.3	3.3
Suspended particles PM-10	19.3	5.3	1.3	0.8	2.0	2.0	12.7	6.7		10.0	6.7	6.0	3.3	4.7	1.9	17.3	0.3	9.3			6.7	16.7	6.5	4.0	11.6	
Suspended particles PM-2.5	13.8	10.8	2.9	0.8	3.1	2.5	7.7	10.8		6.2		12.3		3.4		38.5	0.6	15.4			4.6	9.2	6.9	3.1	2.8	
Hydrogen fluoride											0.3															
Nitrogen dioxide	0.4	3.7	0.6	0.3	0.4	0.4	0.6	1.1	1.4	0.4	1.6	1.3	0.6	0.4	0.4	1.0	0.8	1.2	1.2	0.6	0.6	0.5	1.4	0.9	6.4	0.4
Sulfur dioxide	0.6	1.4	0.7		0.7	0.7	5.3	0.6	2.5	0.8	5.5	0.5	3.0	2.4	0.2	0.7	4.4	3.2	6.8	1.0	0.5	0.4	0.6	2.6	0.2	0.4
Copper	0.5							2.9			0.001						0.02									
Arsenic	25.0							17.5			5.0						1.0									
Ozone (ground level)	0.9			1.6	0.8	0.9	1.6			0.9	1.2	1.0	0.7	0.9	0.7	1.5	0.6	0.6				0.9	0.9	0.9	0.6	
Nitrogen oxide	0.7	0.5	0.9	0.1	0.5	0.5	0.8	1.0	3.1	1.0	2.9	3.2	1.4	0.7	0.4	0.7	0.2	2.8	0.7	1.4	0.6	0.3	2.7	0.4	3.8	0.5
Carbon monoxide	0.6	0.4	0.2		0.2	0.2	1.0	0.9	0.6	0.2	0.7	0.4	0.7	1.0	0.2	3.1	0.7	0.9	1.2	0.3	0.4	0.5	0.8	0.9	0.001	0.7
Hydrogen sulfide	0.3				0.1	0.1	2.4		0.3	1.4	5.0	0.3	0.2	0.3	0.2	0.5	1.8	0.7	1.0		0.01	0.3	0.3	0.3	1.8	
Sulfates		1.4											1.4			0.2	1.2	3.2	0.6			0.6	0.4	0.4	2.6	
Phenol								0.002		0.001	0.01	0.01				0.003		0.009	0.01				0.003		0.3	
Formaldehyde	1.6						3.5	1.02		0.1	1.1		1.0			0.6					0.1				2.1	0.5
Chlorine											0.7												0.1			
Hydrogen chloride											0.1												0.03			
Hydrogen fluoride		0.5											0.1													
Standard HQ ≤ 1.0																										

The hazard quotient results calculated for acute exposure (HQ acute) of the analyzed chemicals, contained in the atmospheric air in the studied cities, are presented in Table 2.

As mentioned above, if HQ is equal to or less than 1.0, the risk of harmful effects is considered extremely low. Therefore, an increase in HQ indicates the probability of the development of harmful effects and potential health risk. Thus, we observed the feasibility of adverse effects (HQ acute) from the different chemical substances in most Kazakhstani cities. The most probable adverse effect associated with ammonia was detected in Temirtau city (1.1) and the least was in Atyrau and Taraz (0.1). No risk was observed in Astana, Kokshetau, Almaty, Kostanay, Kyzylorda and Turkestan.

For suspended particles (dust), the highest risk was detected in Astana (14.7), and the lowest in Petropavlovsk (0.3). There was no risk associated with suspended particles (dust) in Stepnogorsk, Uralsk, or Aksay. The acute exposure hazard quotient for suspended particles PM-10 was highest in Shymkent (19.3) and lowest in Balkhash (0.3). No risks were observed in Taldykurgan, Kostanay, Temirtau, or Turkestan. The corresponding maximum risk for suspended particles PM-2.5 was 38.5 in Karaganda, and 0.6 in Balkhash, constituting the minimum value recorded. PM-2.5 particles were not detected in Taldykurgan, Kostanay, Temirtau, Ust-Kamenogorsk, Aksay, Taraz, or Turkestan.

Hydrogen fluoride was detected only in Ust-Kamenogorsk, with a risk level of 0.3. Nitrogen dioxide and nitrogen oxide were detected in all cities, with the highest risks recorded in Petropavlovsk, 6.4 and 3.8 accordingly. The lowest was in Stepnogorsk with values recorded at 0.3 and 0.1. Sulfur dioxide also was found at high level in almost all the cities. The maximum risk was in Temirtau (6.8) and the minimum was in Aksay and Petropavlovsk (0.2). Copper and arsenic were detected in four cities— Shymkent, Almaty, Ust-Kamenogorsk, and Balkhash—with the maximum risk for copper found in Almaty (2.9), and for arsenic in Shymkent (25).

The risk of ozone (ground level) was approximately the same and varied from 0.6 (Balkhash, Zhezkazgan, Petropavlovsk) to 1.6 (Stepnogorsk, Aktobe).

A high level of carbon monoxide was detected in almost all the cities, but the maximum risk was in Karaganda (3.1), the minimum in Petropavlovsk (0.001). The maximum risk level caused by hydrogen sulfide was detected in Ust-Kamenogorsk (5), the minimum in Kyzylorda (0.01). No risk related to hydrogen sulfide was found in Astana, Kokshetau, Stepnogorsk, Almaty, Kostanay, or Petropavlovsk. The sulfate content of air indicates maximum risk in Zhezkazgan (3.2) and minimum in Karaganda (0.2). The risk of phenol was less than 1 in all cities, with the highest in Petropavlovsk (0.3). Formaldehyde caused maximum risk in Aktobe (3.5), minimum in Atyrau and Kyzylorda (0.1). However, in most of the cities no risk was found. Chlorine and hydrogen chloride were found in the air in only two cities: Ust-Kamenogorsk (0.7 and 0.1) and Pavlodar (0.1 and 0.03). Hydrogen fluoride was present only in Astana (0.5) and Taraz (0.1).

Thus, there is the risk of adverse effects on the population’s health from acute effects of suspended particles, oxides and dioxides of nitrogen, and sulfur in almost all the studied cities. In general, the most unfavorable situation is in Ust-Kamenogorsk, where HQ acute is above 1 with respect to nine chemicals, and for 7 chemical substances in Aktobe, Almaty, and Petropavlovsk. In other cities the HQ acute was above 1 with respect to six substances and below. The risk of adverse effects was determined for only one chemical in Stepnogorsk, Aksay and Turkestan.

It should also be noted that there are no reference concentrations (in case of acute exposure) for some substances (benz(a)pyrene, cadmium, lead, chromium). The concentrations for beryllium during the study period were below the detection limit for the technique used. As a result, it turned out to be impossible to calculate the hazard quotients in case of acute exposure for the above substance.

Then, we calculated the values of hazard quotients for chronic exposure due to the average annual calculated concentration of toxic substances in the surface air of the studied cities. The results are presented in Table 3.

**Table 3:** Hazard quotients of chronic exposure (HQ chronic) due to the main chemical pollutants of the atmospheric air in the studied cities of the Kazakhstan. 1 – Shymkent. 2 – Astana. 3 – Kokshetau. 4 – Stepnogorsk. 5 – Borovoye. 6 – Shchuchinsk-Borovoy resort area. 7 – Aktobe. 8 – Almaty. 9 – Taldykurgan. 10 – Atyrau. 11 – Ust-Kamenogorsk. 12 – Semey. 13 – Taraz. 14 – Uralsk. 15 – Aksay. 16 – Karaganda. 17 – Balkhash. 18 – Zhezkazgan. 19 – Temirtau. 20 – Kostanay. 21 – Kyzylorda. 22 – Aktau. 23 – Pavloda. 24 – Ekibastuz. 25 – Petropavlovsk. 26 – Turkestan.

*Cities	1	2	3	4	5	6	7	8	9	10	11	12	13	14	15	16	17	18	19	20	21	22	23	24	25	26

Substances																										

Ammonia	0.2			0.2	0.1	0.1	0.04		0.1	0.04	0.1	0.1	0.1	0.04	0.03	0.1	0.1		0.6			0.1	0.1	0.1	0.02	
Benz(a)pyrene											700.0		100.0													
Suspended particles (dust)	3.4	4.0	0.8		0.5	0.4	0.4	2.3	0.9	1.8	1.3	1.6	2.0		0.2	1.9	2.3	4.3	3.6		0.8	2.8	1.2	1.7		1.3
Suspended particles PM-10	2.0	1.2	0.04	0.1	0.4	0.4	0.8	0.6		0.4	1.0	0.4	0.8	0.4		2.0	0.6	0.6		0.2	0.8	2.0	0.1	0.2	0.05	
PM-2.5 suspended particles	2.7	1.3	0.1	0.1	1.3	1.3	1.3	0.6		0.7		2.0		0.7		6.7	2.0	0.7			0.7	1.3	0.4	0.7	1.1	
Hydrogen fluoride											0.5															
Nitrogen dioxide	1.0	2.0	0.3	0.1	0.2	0.2	0.5	1.8	1.3	1.0	1.5	0.8	1.8	0.5	0.5	1.3	0.5	1.0	0.5	0.8	1.3	0.5	0.8	0.5	22.5	0.3
Sulfur dioxide	0.2	0.5	0.1		0.5	0.2	0.2	1.1	0.8	0.2	2.2	0.5	0.2	0.3	0.02	0.4	0.6	0.4	1.0	0.5	1.4	0.4	0.3	0.1	0.2	0.2
Cadmium	450.0							50.0			3.0						479850.0									
Manganese													74800.0													
Copper	800.0							4050.0			2.5						22.7									
Methane											0.03	0.03		0.002		0.02	0.01		0.01				0.002	0.01		
Arsenic	166.7							16.7			3.3						271333.3									
Ozone (ground level)	1.9			1.3	0.5	0.6	2.8			1.1	1.4	1.5	1.3	1.0	1.1	0.8	1.2	0.5				1.8	0.7	1.2		
Nitrogen oxide	0.2	0.3	1.8	0.1	0.1	0.1	0.2	0.7	1	0.1	0.3	0.5	0.3	0.2	0.02	0.1	0.03		0.2	0.3	0.2	0.2	0.4	0.1	13.3	0.1
Carbon monoxide	0.7	0.2	0.1		0.1	0.1	0.3	0.3	0.3	0.3	0.3	0.3	0.5	0.1	0.03	0.3	0.3	0.3	0.4	0.2	0.1	0.1	0.2	0.3	0.0001	0.2
Lead	20.0							66.0			0.7		18180.0				1.4									
Hydrogen sulfide	1.0				1.4	0.4	1.0		0.5	2.0	1.5	2.0	0.5	1.5		0.5	0.5	4.5	1.0		0.2	1.5	1.0	0.5	8.0	
Sulfates		0.3											0.6			0.3	0.1	0.4	0.4			0.6	0.1	0.1	0.1	
Phenol								0.3		0.3	0.3	0.7				1.0		1.4	0.1				0.1		0.4	
Formaldehyde	7.4						1.0	4.1		0.6	1.3		2.4			4.2					0.3				3.3	0.1
Chlorine											35.0												1.5			
Hydrogen chloride											1.5												1.1			
Hydrogen fluoride		0.1											0.2													
Chromium	10.0						3.0	60.0									90300.0									
Zinc											1.0															
Standard HQ ≤ 1.0																										

Based on the results provided in Table 3, we conclude that there is a high probability of adverse chronic effects caused by different chemicals. For example, the risk caused by ammonia is less than 1 in all cities, though the maximum was detected in Temirtau (0.6). Benz(a)pyrene was found in abundant quantities only in Ust-Kamenogorsk and Taraz (700 and 100 respectively). Suspended particles (dust) were present in almost all cities, and the risk level varied from 0.2 in Aksay to 4.3 in Zhezkazgan. The highest risk of suspended particles PM-10 was in Shymkent, Karaganda, and Aktau (2), whilst the minimum was observed in Stepnogorsk and Pavlodar (0.1). Suspended particles PM-2.5 posed a health threat in Karaganda (6.7). The lowest PM-2.5 level was found in Kokshetau and Stepnogorsk (0.1). Hydrogen fluoride was present in the air only in Ust-Kamenogorsk, with a risk level of 0.5.

Nitrogen dioxide and nitrogen oxide were present in all cities of Kazakhstan (22.5), with the highest risk in Petropavlovsk (13.3) and the minimum levels in Stepnogorsk (0.1) and Aksay (0.02). Sulfur dioxide was also present in almost all the cities under study, with the highest risk recorded in Ust-Kamenogorsk (2.2) and the lowest in Aksay (0.02). Copper and arsenic were detected in only four cities. The maximum risk for copper in Almaty is 4050 and 271333.3 in Balkhash for arsenic. The highest risk for ozone (ground level) was determined as Aktobe (2.8).

Carbon monoxide was detected in all cities, but the risk was less than 1 and was highest in Shymkent (0.7). The risk level of lead was determined in five cities, with the highest recorded in Taraz(18,180). Hydrogen sulfide was present in many cities. The highest risk was in Petropavlovsk (8). The risk level of sulfates was shown to be less than 1 in all cities. Phenol had an increased risk level only in Zhezkazgan (1.4). The maximum risk level of formaldehyde was in Shymkent (7.4). Chlorine and hydrogen chloride were present in only two cities, Ust-Kamenogorsk (35 and 1.5 respectively) and Pavlodar (1.5 and 1.1). Hydrogen fluoride was presented in two cities – Astana (0.1) and Taraz (0.2). The maximum risk level of chromium was recorded in Balkhash, (90,300) and Almaty (60). Zinc was present only in Ust-Kamenogorsk, with a risk level of 1.

Thus, there is risk of adverse effects caused by chronic exposure to suspended particles in the majority of the studied cities. As for the number of chemical substances with increased risk of chronic exposure, the value was maximal in Ust-Kamenogorsk (13), similar to acute exposure. Extremely high HQ of chronic effect as result of exposure to heavy metals was detected in Shymkent, Almaty, Taraz, and Balkhash, and in Ust-Kamenogorsk and Taraz cities for benz(a)pyrene.

It is known that atmospheric air content is the leading environmental factor associated with the majority of health risks. A significant number of large industrial complexes in cities, thermal power plants, coal and other industries pose a constant danger on the human body due to the acute and chronic effects of air pollutants.

It was determined that the overwhelming majority of chemicals with hazard quotient (HQ) in excess, in relation to both chronic and acute exposure, mainly impact the respiratory system (such as nitrogen dioxide, suspended particles, ozone, sulfur dioxide, phenol, formaldehyde, etc.). There was enough HQ data to calculate the hazard indices. Based on the aforementioned, we calculated hazard indices according to their mode of action only for the respiratory system. The hazard indices for chronic and acute effects in the studied cities are presented in Table 4.

It was found that the highest hazard index of acute exposure was observed in Karaganda (HI acute 63.5), followed by Zhezkazgan (44.1) and Shymkent (41.6). The least occurred in Stepnogorsk (3.8), Aksay (4.4), and Turkestan (5.1).

**Table 4:** Hazard index for chronic and acute exposure (HI acute/HI chronic) of the respiratory organs to the main chemical pollutants of the atmospheric air in the studied cities of Kazakhstan.

No	Cities	HI acute	HI chronic

1	Shymkent	41.6	1,446.6
2	Astana	38.3	9.8
3	Kokshetau	10.1	3.2
4	Stepnogorsk	3.8	1.9
5	Borovoye	8.9	5.0
6	Shchuchinsk-Borovoye resort area	9.2	3.7
7	Aktobe	36.7	11.3
8	Almaty	26.3	4,187.8
9	Taldykorgan	11.6	4.5
10	Atyrau	24.8	7.9
11	Ust-Kamenogorsk	30.0	57.5
12	Semey	27.2	9.3
13	Taraz	18.9	74,810.2
14	Uralsk	13.0	4.5
15	Aksay	4.4	1.9
16	Karaganda	63.5	18.3
17	Balkhash	18.5	841,514.1
18	Zhezkazgan	44.1	12.4
19	Temirtau	15.3	7.3
20	Kostanay	6.9	1.7
21	Kyzylorda	16.4	5.6
22	Aktau	30.7	11.2
23	Pavlodar	23.6	7.7
24	Ekibastuz	15.5	5.1
25	Petropavlovsk	32.5	48.6
26	Turkestan	5.1	2.0
Standard HI ≤ 1.0			

The highest hazard index of chronic exposure was observed in Balkhash (HI chronic 841,514.1). This was followed by Taraz (74,810.2), Almaty (4,187.8), and Shymkent (1,446.6). The least was in Kostanay (1.7), followed by Stepnogorsk (1.9) and Aksay (1.9).

Extremely high hazard indices of the chronic exposure were a cause for attention in Balkhash, Taraz, Almaty, and Shymkent. At the same time, the hazard indices of acute exposure in these cities were at the average levels, except for Shymkent.

Considering the fact that there are high rates of cancer incidence in the regions of Kazakhstan, coupled with research results showing the existence of adverse risk effects in practically every inhabited locality studied, caused by the chronic exposure of chemical substances, we calculated the individual carcinogenic risk (ICR) presented in the Table 5.

**Table 5:** Individual carcinogenic risk (ICR) in the studied cities of the Kazakhstan.

Cities	Shymkent	Aktobe	Almaty	Atyrau	Ust-Kamenogorsk	Taraz	Karaganda	Balkhash

Substances								

Cadmium	1.62E-02		1.80E-03		1.08E-04			1.73E+01
Copper								
Arsenic	2.14E-02		2.14E-03		4.29E-04			3.49E+01
Carbon monoxide								
Lead	1.20E-04		3.96E-04		4.32E-06	1.09E-01		8.40E-06
Formaldehyde	2.93E-04	3.94E-05	1.62E-04	2.50E-05	5.26E-05	9.46E-05	1.64E-04	
Chromium	1.20E-02	3.60E-03	7.20E-02					1.08E+02

According to the criteria of carcinogenic risk assessment, unacceptable risk levels have been determined for professional groups and the whole population with respect to cadmium in Shymkent, Almaty, and Balkhash; arsenic in Shymkent, Almaty, and Balkhash; lead in Taraz; chromium in Shymkent, Aktobe, Almaty, and Balkhash.

The acceptable carcinogenic risk level for professional groups, but unacceptable for the population was determined for cadmium and arsenic in Ust-Kamenogorsk; for lead, in Shymkent and Almaty; for formaldehyde, in Shymkent, Almaty, and Karaganda.

Thus, in the listed cities the unacceptable carcinogenic risk level for the population is identified. High rates of ICR do not guarantee the incidence of cancer, but only increase its probability. It requires urgent management decisions to eliminate and/or reduce the risk levels.

## Study limitations

Before interpreting the quantitative risk assessment results obtained above, it is necessary to take into account study limitations. Risk assessment was carried out only according to the official data of KAZHYDROMET regional state enterprise, based on the analysis and processing of air samples taken at the stationary observation posts. Average daily measurements were carried out according to short (two times a day), incomplete (three times a day) and complete (four times a day) programs, i.e. the measurements were averaged with no more than 6-hour intervals. According to Directive No. 2008/50/EC—Atmospheric air quality and measures for its purification—adopted by the European Parliament and the Council of the European Union, when determining the maximum permissible level of chemical substances to protect the human health, a reliable data ratio of 75% of the one-hour value is required, i.e. 45 minutes. For a 24-hour value (average daily), 75%, i.e. at least, 18 average hourly values. It means that for the most objective risk assessment it is necessary to take into account not less than 18 averaged one-hour values for the average daily measurement.

Thus, the maximum one-time and average daily measurements conducted by the regional state enterprise KAZHYDROMET at the stationary posts, even according to the full program, may not reflect the actual atmospheric air condition, which may affect the quantitative risk assessment results. In this regard, there is a need to study the monitoring data of alternative sources and to carry out data collection in accordance with the regulations of the European Union Directive No. 2008/50/EC on data collection rules for statistical processing.

## Conclusion

Atmospheric air quality analysis in the main cities of Kazakhstan in context of its impact on the health of the populace was carried out. A public health risk assessment based on the measurement data analysis of the atmospheric air quality was conducted. The following conclusions can be made from the results:

First of all, it should be noted that the values of the hazard quotients and indices for acute and chronic exposure in most of the studied cities of the Republic of Kazakhstan exceed the permissible level equal to 1.0.

### Acute risk effects

There are acute risk effects on the health of the populace in the studied cities of Kazakhstan, due to air pollution by the following pollutants: suspended particles, oxides and dioxides of nitrogen and sulfur, and heavy metals (copper and arsenic). Generally, the most dangerous situations are in Ust-Kamenogorsk, Shymkent, Aktobe, Almaty, and Petropavlovsk. Stepnogorsk, Aksay, and Turkestan have the most favorable ecological condition.

### Chronic risk effects

There are also adverse risk effects caused by chronic exposure of suspended particles in majority of the studied cities, as well as adverse effects of benz(a)pyrene, nitrogen dioxide and nitrogen oxide, chlorine, and heavy metals (cadmium, manganese, copper, arsenic, lead, and chromium). The maximum chronic exposure risk is in Ust-Kamenogorsk. The least risk level is in Kokshetau, Stepnogorsk, the Shchuchinsk-Borovoye resort area, Taldykorgan, Uralsk, Aksai, Temirtau, Ekibastuz, and Turkestan cities. It is important to note the extremely high HQ of chronic effect caused by the heavy metals in Shymkent, Almaty, Taraz, and Balkhash, as well as for benz(a)pyrene in Ust-Kamenogorsk and Taraz cities.

### Hazard index analysis

High hazard indices for the respiratory system were detected. It was revealed that the highest hazard index of acute exposure for respiratory system is in Karaganda, Zhezkazgan, and Shymkent; the least in Stepnogorsk, Aksay, and Turkestan. The maximum hazard index of chronic exposure of the respiratory system to air pollutants is in Balkhash, Taraz, Almaty, and Shymkent; the minimum in Kostanay, Stepnogorsk, and Aksay. Paying attention to this fact, we also consider it necessary in the future to calculate the HI for cardiovascular and central nervous systems.

### Carcinogenic risk

In addition, the carcinogenic risk level both for professional groups and the whole population represents a great danger, because it is defined as unacceptable in Shymkent, Almaty, Balkhash, Aktobe, Taraz, and Ust-Kamenogorsk cities.

### Recommendations

In line with the aforementioned, it is strongly recommended that due to the unacceptable risk level, it is necessary to immediately conduct a detailed study of the health status of the population, depending on the air pollution in the cities with high risk levels. Additionally, the research results indicate that it is obligatory to develop management decisions so as to reduce the risk levels.

## References

[1] Bergstra AD, Brunekreef B, et al. The mediating role of risk perception in the association between industry-related air pollution and health. Plos One. 2018; 13(5): e0196783 DOI: 10.1371/journal.pone.0196783 29723277 PMC5933722

[2] Brauer M, Freedman G, et al. Ambient air pollution exposure estimation for the global burden of disease 2013. Environmental Science & Technology. 2016; 50(1): 79–88. DOI: 10.1021/acs.est.5b03709 26595236

[3] de la Gala Morales M, Holgado FR, et al. Ambient air levels and health risk assessment of benzo(a)pyrene in atmospheric particulate matter samples from low-polluted areas: Application of an optimized microwave extraction and HPLC-FL methodology. Environmental Science and Pollution Research International. 2015; 22(7): 5340–5349. DOI: 10.1007/s11356-014-3722-x 25345924

[4] Feng J, Yu H, et al. PM_2.5_ levels, chemical composition and health risk assessment in Xinxiang, a seriously air-polluted city in North China. Environmental Geochemistry and Health. 2017; 39(5): 1071–1083. DOI: 10.1007/s10653-016-9874-5 27613015

[5] Koman PD, Hogan KA, et al. Examining joint effects of air pollution exposure and social determinants of health in defining “at-risk” populations under the clean air act: susceptibility of pregnant women to hypertensive disorders of pregnancy. World Medical & Health Policy. 2018; 10(1): 7–54. DOI: 10.1002/wmh3.257 30197817 PMC6126379

[6] Lee JY and Kim H. Ambient air pollution-induced health risk for children worldwide. The Lancet. Planetary Health. 2018; 2(7): e285–e286. DOI: 10.1016/S2542-5196(18)30149-9 30074888

[7] Liu X, Zhang H, et al. A novel computational solution to the health risk assessment of air pollution via joint toxicity prediction: A case study on selected PAH binary mixtures in particulate matters. Ecotoxicology and Environmental Safety. 2019; 170: 427–435. DOI: 10.1016/j.ecoenv.2018.12.010 30553920

[8] Liu X, Zhu H, et al. Public’s health risk awareness on urban air pollution in Chinese megacities: The cases of Shanghai, Wuhan and Nanchang. International Journal of Environmental Research and Public health. 2016; 13(9). DOI: 10.3390/ijerph13090845 PMC5036678 27571088

[9] Lorber M. Indirect exposure assessment at the United States Environmental Protection Agency. Toxicology and Industrial Health. 2001; 17(5–10): 145–156. DOI: 10.1191/0748233701th113oa 12539859

[10] Marth E, Haselbacher-Marko S, et al. A cohort study with children living in an air-polluted region—a model for public health. Toxicology Letters. 1996; 88(1–3): 155–159. DOI: 10.1016/0378-4274(96)03731-9 8920730

[11] Morelli X, Rieux C, et al. Air pollution, health and social deprivation: a fine-scale risk assessment. Environmental Research. 2016; 147: 59–70. DOI: 10.1016/j.envres.2016.01.030 26852006

[12] Orru K, Nordin S, et al. The role of perceived air pollution and health risk perception in health symptoms and disease: A population-based study combined with modelled levels of PM_10_. International Archives of Occupational and Environmental Health. 2018; 91(5): 581–589. DOI: 10.1007/s00420-018-1303-x 29602966 PMC6002462

[13] Sanhueza PA, Torreblanca MA, et al. Particulate air pollution and health effects for cardiovascular and respiratory causes in Temuco, Chile: a wood-smoke-polluted urban area. Journal of the Air & Waste Management Association. 2009; 59(12): 1481–1488. DOI: 10.3155/1047-3289.59.12.1481 20066914

[14] Schlesinger RB and Graham JA. Health effects of atmospheric acid aerosols: a model problem in inhalation toxicology and air pollution risk assessment. Fundamental and Applied Toxicology: Official Journal of the Society of Toxicology. 1992; 18(1): 17–24. DOI: 10.1016/0272-0590(92)90190-S 1601205

[15] Shaddick G, Thomas ML, et al. Data integration for the assessment of population exposure to ambient air pollution for global burden of disease assessment. Environmental Science & Technology. 2018; 52(16): 9069–9078. DOI: 10.1021/acs.est.8b02864 29957991

[16] Surzhikov VD, Surzhikov DV, et al. Atmospheric air pollution in an industrial city as the factor of non-carcinogenic risk for health of communities. Gigiena i Sanitariya. 2013; 1: 47–49. 23805694

[17] Suter GW. Ecological risk assessment in the United States Environmental Protection Agency: A historical overview. Integrated Environmental Assessment and Management. 2008; 4(3): 285–289. DOI: 10.1897/IEAM_2007-062.1 18321143

